# Systemic vs. Topical Therapy for the Treatment of Vulvovaginal Candidiasis

**DOI:** 10.1155/S1064744994000098

**Published:** 1994

**Authors:** Sebastian Faro

**Affiliations:** Department of Gynecology and Obstetrics University of Kansas School of Medicine 3901 Rainbow Boulevard Kansas City KS 66160-7316 USA

## Abstract

It is estimated that 75% of all women will experience at least 1 episode of vulvovaginal candidiasis (VVC) during their lifetimes. Most patients with acute VVC can be treated with short-term regimens that optimize compliance. Since current topical and oral antifungals have shown comparably high efficacy rates, other issues should be considered in determining the most appropriate therapy. It is possible that the use of short-duration narrow-spectrum agents may increase selection of more resistant organisms which will result in an increase of recurrent VVC (RVVC). Women who are known or suspected to be pregnant and women of childbearing age who are not using a reliable means of contraception should receive topical therapy, as should those who are breast-feeding or receiving drugs that can interact with an oral azole and those who have previously experienced adverse effects during azole therapy. Because of the potential risks associated with systemic treatment, topical therapy with a broad-spectrum agent should be the method of choice for VVC, whereas systemic therapy should be reserved for either RVVC or cases where the benefits outweigh any possible adverse reactions.


Infectious Diseases in Obstetrics and Gynecology 1:202–208 (1994)
© 1994 Wiley-Liss, Inc.

### Systemic vs. Topical Therapy for the Treatment of Vulvovaginal Candidiasis

**Sebastian Faro**

*Department of Gynecology and Obstetrics, University of Kansas School of Medicine, Kansas City, KS*

Abstract

**It is estimated that 75% of all women will experience at least 1 episode of vulvovaginal candidiasis (VVC) during their lifetimes. Most patients with acute VVC can be treated with short-term regimens that optimize compliance. Since current topical and oral antifungals have shown comparably high efficacy rates, other issues should be considered in determining the most appropriate therapy. It is possible that the use of short-duration narrow-spectrum agents may increase selection of more resistant organisms which will result in an increase of recurrent VVC (RVVC). Women who are known or suspected to be pregnant and women of childbearing age who are not using a reliable means of contraception should receive topical therapy, as should those who are breast-feeding or receiving drugs that can interact with an oral azole and those who have previously experienced adverse effects during azole therapy. Because of the potential risks associated with systemic treatment, topical therapy with a broad-spectrum agent should be the method of choice for VVC, whereas systemic therapy should be reserved for either RVVC or cases where the benefits outweigh any possible adverse reactions.** © 1994 Wiley-Liss, Inc.

Key words

Adverse effects, compliance, drug interactions, oral therapy, pregnancy, topical therapy

Vulvovaginal candidiasis (VVC) remains, after bacterial vaginosis, the most common vaginal infection in the United States.[1,2] Researchers estimate that 75% of all women will have 1 VVC infection during their lifetimes, typically during their childbearing years.[1,3,4] Between 40% and 50% of women who have experienced 1 episode of VVC are likely to have at least 1 recurrence, and approximately 5% will have repeated episodes of the disorder.[1]

Of particular concern to physicians and other health care professionals is the fact that the number of prescriptions written in the United States to treat yeast infections has almost doubled between 1980 and 1990, reaching 13 million.[1] Statistical data derived from patients treated at genitourinary medicine centers in the United Kingdom indicate a similarly sharp increase, from 118/100,000 to 200/100,000 patients during the last decade. Moreover, although the causative organism in 85–90% of all cases of VVC is *Candida albicans*, some evidence suggests that the proportion of non-*albicans Candida* infections, including those caused by *C. (Torulopsis) glabrata*, *C. tropicalis*, and *C. krusei*, is rising substantially.[2,5] Traditional imidazole treatment regimens have often shown less efficacy against those species than against most of the approximately 200 known pathogenic strains of *C. albicans*.[1,6,7]

Amid these causes for concern, however, positive developments are the number of therapeutic agents and range of treatment options available for the management of VVC. Most women with acute VVC can be treated with short-term regimens that are likely to enhance compliance and thus minimize the risk of treatment failure.[2,8,9] Newer triazole

Address correspondence/reprint requests to Dr. Sebastian Faro, Department of Gynecology and Obstetrics, University of Kansas School of Medicine, 3901 Rainbow Boulevard, Kansas City, KS 66160-7316.

Received February 16, 1994
Accepted February 22, 1994

Review Article

**TABLE 1. Drug formulations and doses commonly used to treat VVC[a]**

| Agent | Formulation | Dose |
|---|---|---|
| Nystatin | 100,000 U vaginal tablet | 100,000 U × 14 days |
| Clotrimazole | 1% cream | 5 g × 7–14 days |
| | 100 mg vaginal tablet | 100 mg × 7 days |
| | 100 mg vaginal tablet | 200 mg × 3 days |
| | 500 mg vaginal tablet | 500 mg × 1 dose |
| Miconazole | 2% cream | 5 g × 7 days |
| | 100 mg vaginal suppository | 100 mg × 7 days |
| | 200 mg vaginal suppository | 200 mg × 3 days |
| | 1,200 mg vaginal suppository | 1,200 mg × 1 dose |
| Tioconazole | 2% cream | 5 g × 3 days |
| | 6.5% cream | 5 g × 1 dose |
| Econazole | 150 mg vaginal tablet | 150 mg × 3 days |
| Terconazole | 0.4% cream | 5 g × 7 days |
| | 0.8% cream | 5 g × 3 days |
| | 80 mg vaginal suppository | 80 mg × 3 days |
| Ketoconazole | 200 mg oral tablet | 400 mg × 5 days[32] |
| Fluconazole | 150 mg oral capsule | 150 mg × 1 dose[25] |
| Itraconazole | 100 mg oral capsule | 200 mg × 3 days[14] |

[a]Adapted in part from Sobel.[1]

formulations provide a broader spectrum of activity against non-*albicans* species, thereby suggesting the possibility of better cure rates and less recurrent disease. One decision for the physician, given this diversity of options, is whether to prescribe a topical or an oral agent for the patient with VVC.

This review discusses the potential benefits and liabilities of topical vs. systemic (oral) drug therapy, taking into account such factors as efficacy in acute and recurrent VVC (RVVC), potential for the development of resistance, pathogen selection or shift, VVC during pregnancy, potential for drug interactions, safety profiles, and patient acceptance. Table 1 summarizes the formulations and dosages of those agents that are commonly used to treat VVC.

### TREATMENT OF TOPICAL INFECTIONS WITH TOPICAL PREPARATIONS

As a rule, it is preferable to treat topical disorders with topical therapy. It is always advisable to involve the smallest possible number of body systems in the pharmacotherapeutic process. Even with the use of a systemic agent that has a good safety profile, concern about the development of potentially serious adverse effects remains. In an era of fiscal constraint, when the effective use of physician time and the reduction of patient costs are regarded as essential, the unnecessary monitoring of patients for untoward effects of systemic therapy and the possible need for additional physician visits are difficult to defend.

Concern about adverse effects is compounded when therapy focuses on women of childbearing age whose acute or recurrent disorder may be an unrecognized sign of pregnancy. Whether or not preclinical investigations and existing data from human populations have suggested that a given systemic agent has the potential for teratogenesis, unless the absence of pregnancy is confirmed, the prudent course is to treat the patient who could be pregnant with a topical agent that has a well-established reproductive health profile.

Similarly, the risk of potentially serious drug-drug interactions always remains when a systemic drug is used in lieu of a topical agent. This risk is significantly increased when a systemic agent is to be used primarily in a population whose members are known to make extensive use of an interacting class of drugs, i.e., oral contraceptives. Even when the physician clearly delineates the implications of drug-drug interactions to a patient and explicitly questions her about current use of potentially interacting agents, full disclosure by the patient cannot be guaranteed.

Many studies have shown that good compliance is unlikely to be achieved unless the patient regards the presenting problem as a severe disorder. Despite VVC's potential seriousness, women tend to view it as a nuisance or an inconvenience rather than a major health problem. Thus, it cannot be assumed that patients who use a systemic agent will refrain from the concurrent use of potentially interacting drugs or modify their behaviors to minimize the risks of drug-drug interactions.

### EFFICACY IN ACUTE VVC

In immunocompetent patients with acute VVC, the overall clinical and mycological cure rate is extremely high with both topical and oral agents.[1,10] The forms of topical antimycotic drugs include creams, lotions, vaginal tablets, and suppositories. There are no data that the formulation of a topical agent influences its clinical efficacy or that the majority of women prefer a particular form of local administration.[1] With nystatin, the average myco-

**TABLE 2. Risk factors for VVC[1,13]**

| |
|---|
| **Predisposing conditions** |
| Pregnancy, especially in the 3rd trimester |
| Diabetes mellitus that is poorly controlled |
| Cushing's disease |
| Addison's disease |
| Hypo- or hyperthyroidism |
| Debilitating disease, e.g., leukemia, severe vitamin deficiency |
| AIDS or HIV infection |
| Vaginal trauma |
| **Predisposing practices** |
| Use of high-estrogen oral contraceptives |
| Use of antibiotics, especially tetracycline, ampicillin, and cephalosporins |
| Use of therapies that compromise the immune system, including hormones, cytotoxic agents, immunosuppressive drugs, and radiotherapy |
| Frequent, traumatic sexual intercourse |
| Wearing of tight clothing or nylon underwear |
| Use of intrauterine contraceptive devices |
| Ingestion of a large amount of candy |

logical cure rate is generally considered to range from 75% to 80%, while it is estimated to be somewhat higher, from 85% to 90%, with the azole derivatives.[1,3] Because of its broader spectrum of activity, the triazole terconazole has shown excellent efficacy against a wide range of pathogenic fungi.[3,8]

### EFFICACY IN RVVC

RVVC, the occurrence of at least 4 mycologically proven symptomatic episodes of VVC in 1 year, occurs in approximately 5% of patients with VVC.[11] Although it occurs at extremely high rates among immunocompromised women, including approximately 24% of human immunodeficiency virus (HIV)-infected patients,[12] it is also found in women who have none of the risk factors generally associated with VVC (Table 2).[1,11,13] The cause of RVVC has not been determined, but several explanations have been proposed.

RVVC has often been attributed to the existence of an intestinal reservoir of *Candida* that acts as a source of reinfection.[2,11] However, while it is true that some patients with RVVC demonstrate rectal colonization by *Candida* species, such colonization has also been found in approximately 40% of asymptomatic women. Furthermore, biotyping of vaginal and rectal cultures simultaneously obtained from patients with RVVC has often shown a low concordance between cultures. A longitudinal study of women with RVVC who were receiving ketoconazole therapy showed that VVC sometimes recurred when rectal cultures were negative for *Candida* species.[1] In addition, treatment with nystatin, which reduces intestinal yeast carriage, has failed to prevent symptomatic RVVC. Although the clinical relevance of existing *Candida* reservoirs remains open to investigation, systemic oral therapy may be used as an effective means of destroying these reservoirs.[10,14,15]

Most patients with RVVC require long-term maintenance regimens with mycosuppressive prophylaxis. Extended therapy with topical clotrimazole or miconazole or with oral ketoconazole has been shown to significantly reduce the frequency of RVVC for the duration of treatment.[11] Nevertheless, approximately 50% of patients experience a relapse within several months after treatment ends.[1,11] Although some investigators have suggested that oral therapy is more likely than vaginal therapy to promote the necessary level of patient compliance during long-term treatment,[1,9] others have argued that the greater risk of systemic toxicity associated with oral agents points in favor of topical therapy for RVVC.[16] Oral therapy has the potential to increase the amount of physician time devoted to patient monitoring and to increase patient expenses associated with physician visits and laboratory tests.

### RESISTANCE AND PATHOGEN SHIFT

Some controversy exists as to whether the trend toward shorter courses of therapy for acute VVC and the use of long-term maintenance regimens in RVVC have led to the selection of more resistant strains of *Candida* species, such as *C. tropicalis*, *C. krusei*, and *C. glabrata* (formerly *Torulopsis glabrata*).[11,2] It has been argued that, although *C. albicans* remains the most common cause of VVC, other, more resistant species are gradually assuming a more prominent role. In examining the results of 9 studies conducted in the 1970s, Horowitz et al.[2,5] found that the prevalence of non-*albicans* species approximated 9.9%. By the 1980s, 7 studies revealed a non-*albicans* prevalence of 21.3%. During that period, the incidence of *C. glabrata* was found to have increased from 4.6% to 6.7%, while that of *C. tropicalis* rose from 1.3% to 8.2%. Although the phenomenon of pathogen shift among yeasts has not been definitively proven,[11] most data seem to point in that direction.

### Resistance to Topical Agents

Both in vivo and in vitro studies have demonstrated the lack of development of resistance to topical antifungal agents.[8] One barrier to resistance may be the high drug concentration attained at the site of infection. This is especially true of the azole derivatives, which act on multiple targets on the plasma membrane and within the yeast cell. The only available topical triazole, terconazole, has the added benefit of a broad spectrum of activity against many non-*albicans Candida* species, which may prevent selection of more resistant yeasts.[3,8]

### Resistance to Oral Agents

Among the 3 oral azole antifungal agents, the frequency with which resistance has been described in a clinical setting differs considerably.[12,17] Fluconazole has been implicated in emergent resistance more frequently than ketoconazole, and ketoconazole more often than itraconazole. Although most reported cases of resistance have involved long-term administration to immunocompromised patients with sexually transmitted diseases or neutropenia, the development of resistance may also have some correlation in the treatment of VVC. For example, in patients with acquired immune deficiency syndrome (AIDS) and neutropenia and those receiving treatment in intensive care units, *C. glabrata* and *C. krusei* are frequently cited as emergent colonizing flora during frequent or sustained fluconazole therapy. Those species have also been associated with treatment failures in patients with disseminated infections and mucosal *Candida* infections. Odds[17] observes that, although most of these treatment failures are probably related to the limited susceptibility of *C. glabrata* and *C. krusei* to fluconazole, the development of secondary resistance by *C. glabrata* strains has also been described. Del Palacio[18] has observed that, although empiric prophylactic use of fluconazole in neutropenic patients has led to a decreased frequency of *C. albicans* and *C. tropicalis* infections, such therapy seems to encourage the emergence of less common strains with a native resistance to fluconazole, including *C. krusei, C. glabrata, C. lambica,* and *C. lusitaniae.*

### MANAGEMENT OF VVC DURING PREGNANCY

During pregnancy, the vagina is particularly susceptible to *Candida* infection because of a higher glycogen content, increased estrogen levels, and the presence of other reproductive hormones.[1] *Candida* strains can be found in vaginal cultures of 10–20% of pregnant women, and the incidence of colonization has been reported to be even higher during the 3rd trimester of pregnancy.[10] The incidence of symptomatic VVC is twice as high as it is in the nonpregnant state. Treatment of VVC during pregnancy is indicated not only to alleviate the woman's symptoms, but also to protect the fetus from potentially fatal *Candida* sepsis.[10]

Although clinical response during pregnancy tends to be slower and recurrences are more frequent, most topical antifungal agents are effective, especially when administered for 1–2 weeks.[1] Although little systemic absorption of topical antifungal drugs occurs, the risk to the fetus during the 1st trimester of pregnancy should be weighed against the benefit derived by the mother. Many practitioners consider treatment with topical agents to be relatively safe during the 2nd and 3rd trimesters.[9] Administration of oral ketoconazole, fluconazole, and itraconazole during pregnancy is not recommended.[19,20] Because the development of VVC may be an early sign of pregnancy and since it is not always feasible to administer a pregnancy test to each woman of childbearing potential who presents to the physician with acute VVC, the safest course is the use of a topical agent for first-line therapy.

In addition, fluconazole, itraconazole, and probably ketoconazole are excreted in breast milk. Therefore, the use of systemic agents should be limited to those women who choose not to breastfeed. Alternately, topical therapy can be used during this time.

### POTENTIAL FOR DRUG INTERACTIONS

Although significant drug interactions have not been associated with the use of topical antifungal agents, the potential for such drug interactions exists with oral therapy.[21] Table 3 lists those agents that are associated with the greatest risk for significant interaction with ketoconazole, fluconazole, and itraconazole.

Among the drug interactions associated with administration of the oral azoles, 2 are of particular concern because of the characteristics of the patients who typically present with VVC. It is ironic that, although VVC generally occurs in women of childbearing age and is especially common among those

**TABLE 3. Agents with the greatest potential for interaction with the oral azoles[27–29]**

| | |
|---|---|
| Antacids | Isoniazid |
| Anticholinergics | Loratadine |
| Anticoagulants | Methylprednisolone |
| Astemizole | Oral contraceptives |
| Cimetidine | Phenytoin |
| Cyclosporine | Potassium |
| Digoxin | Rifampin |
| Diuretics | Sulfonylureas |
| $H_2$ antagonists | Terfenadine |
| Hypoglycemics | |

who take estrogen-containing oral contraceptives,[1] the oral azoles have the potential to interact with oral contraceptives.[22] As noted previously, the oral azoles are not recommended for young women who are not using a reliable method of birth control.[10]

Another area of special concern is that of diabetic patients. Although women with diabetes are at heightened risk for the development of VVC,[1,13] the potential for interaction exists when oral azoles are concurrently taken with oral hypoglycemics.[23] The fact that a diabetic woman has developed symptomatic VVC suggests that her blood glucose levels are poorly controlled, and treatment of VVC with an oral azole can further compromise that control.

In view of the increasing use of the nonsedating antihistamines terfenadine and astemizole, physicians must advise patients of the severe cardiovascular adverse effects, including prolonged QT intervals, that may arise when oral azoles are coadministered with those agents.[23] Conversely, patients should be cautioned that concomitant use of $H_2$ antagonists can impair the efficacy of the oral azoles.

The extent to which drug interactions with oral azoles may occur in actual clinical practice was suggested by a cross-sectional observational study conducted over a 12-week period at an urban hospital in Australia.[23] Of 76 patients receiving fluconazole, 35 had been prescribed potentially interacting drugs and 9 of those patients were judged to have experienced 10 possible adverse interactions. In 1 patient who received concomitant cyclosporine and fluconazole, the cyclosporine plasma concentration rose from 753 ng/ml to 1,543 ng/ml after 6 days of combination therapy. Potential adverse reactions also occurred in patients receiving fluconazole in conjunction with the following drugs: warfarin (2 of 4 patients), theophylline (1 of 2), phenytoin (3 of 9), rifabutin (2 of 4), and rifampin (1 of 5).

The risk of potentially serious drug interactions may place a significant burden on the physician who elects to prescribe one of the oral azoles as first-line therapy for VVC. Patients must be questioned carefully about the medications they are currently receiving, and the possibility of an inaccurate self-report always exists. The physician who consciously prescribes an oral azole for a woman who is also receiving a potentially interacting drug must expend significant effort in monitoring the patient. Similarly, the patient may be required to bear the economic burden of additional physician visits and laboratory tests to monitor plasma concentrations or other relevant parameters.

### ADVERSE EFFECT PROFILES

Overall, a low incidence of adverse effects has been reported for both topical and oral antifungal agents used to treat VVC.[1] It is noteworthy that a similar incidence and range of adverse effects have sometimes been reported by VVC patients receiving active drug and placebo.[14]

### Adverse Reactions and Topical Therapy

Administration of topical azoles is associated with few local and systemic side effects.[1] The most common local effects, which are usually minor, are vaginal burning, stinging, itching, irritation, and pain; dyspareunia may also occur.[3,8,14,24,25] Overall, approximately 7% of women using azole antifungal cream preparations have reported some degree of treatment-related vaginal discomfort.[9] Additional side effects reported with topical agents include increased frequency of urination, pelvic cramps, dysmenorrhea, paresthesia, rhinorrhea, headache, fever, and chills.[3,8,15,22,24,25]

### Adverse Effects Associated With Ketoconazole

The most common adverse effects reported with ketoconazole are mild gastrointestinal disorders, including nausea, vomiting, and abdominal pain; these have been reported in approximately 10% of patients receiving the drug.[1] At doses greater than 400 mg/day, some cases of androgen biosynthesis blockade have been described.[20] A few cases of anaphylaxis have also been reported. Although hepatotoxicity was initially estimated to occur in

1/10,000 patients treated, the reported incidence has more recently fallen to 1/70,000. Part of this decrease can probably be attributed to a tendency for physicians to prescribe shorter courses of therapy. The incidence of hepatic reactions appears to be higher in women; patients being treated for onychomycosis; patients with preexisting liver disease or alcoholism; patients who receive more than 2 weeks of therapy or who have previously received griseofulvin; and those over the age of 50 years.[16]

### Adverse Effects Associated With Fluconazole

Mild gastrointestinal symptoms, including nausea, vomiting, abdominal pain, and diarrhea, have been reported in approximately 5% of patients receiving fluconazole for VVC.[4,18,25,26] Headache, dizziness, dry mouth, rash, and dry skin have also been reported with some regularity. Approximately 5.1% of patients treated with fluconazole for superficial infection show abnormal liver function test results, as opposed to 3.7% of patients receiving topical treatments with clotrimazole.[20]

Although severe toxic epidermal necrolysis has been reported in a small number of patients treated with fluconazole, those patients were typically AIDS patients who had been receiving concomitant drug therapy for tuberculosis. Thus, a clear association between fluconazole and epidermal necrolysis has not been established.[20] One case of thrombocytopenia has also been reported. A case of angioedema with buccal ulceration was reported in a 21-year-old woman who had taken the 6th of twice-monthly prophylactic doses of 150 mg fluconazole that had been prescribed for RVVC of 3 years duration.[27]

The case of a healthy 22-year-old woman who experienced an anaphylactic reaction a few minutes after administration of 150 mg of oral fluconazole as treatment for VVC has been reported.[28] Although the patient had had no previous contact with fluconazole, she had been treated with ketoconazole 2.5 years before the reported reaction. She had also received vaginal metronidazole 1 year before and oral metronidazole 1 week before the event. Results of a prick test with fluconazole solution were positive at a 1:10 dilution. It appears likely that the patient experienced an allergic reaction to fluconazole that may have been caused by cross-sensitization to ketoconazole or metronidazole.

### Adverse Effects Associated With Itraconazole

The overall incidence of minor adverse effects reported with itraconazole has been estimated to range from 7% to 10%.[20] Most have consisted of mild gastrointestinal disturbances.[13] Malaise, headache, dizziness, transient faintness, psychiatric effects, fever, hypertension, and edema have also been reported.[14,16,22] Hypokalemia has been associated with high-dose therapy. Although a few cases of reversible peripheral neuropathy have been reported, a firm association with itraconazole has not been established.[20] The incidence of hepatic reactions, including hepatitis, is approximately 6 in almost 5 million treatments. A low frequency (0.9%) of reversible changes in liver function tests has also been reported.[13,14,20]

### PATIENT ACCEPTANCE

Since acute VVC in immunocompetent patients usually responds to antifungal treatment, it has often been argued that many apparent treatment failures are actually the results of poor patient compliance.[9,15,24] Because studies have repeatedly shown that key factors in achieving compliance are a brief, uncomplicated dosage regimen and the patient's satisfaction with the nature of the treatment, patient preferences are important considerations in evaluating treatment alternatives.[1] Some studies have indicated that when efficacy is comparable, women tend to prefer oral to topical therapy.[29,30] It has also been suggested, however, that application of a topical agent to the affected area may confer more rapid symptomatic relief for some patients. Whether this benefit is real or only perceptual, the physician may wish to consider concomitant therapy with a topical drug when using a systemic antifungal.

### CONCLUSIONS

In light of the comparable levels of efficacy reported for topical and oral agents in the treatment of VVC and RVVC, major criteria for determining whether to prescribe a local or systemic agent include issues related to drug resistance and pathogen shift, safety in pregnancy, drug interactions, adverse effects, and patient acceptance.

Patients who are pregnant or suspected to be pregnant and women of childbearing age who are not using a reliable method of contraception should receive topical therapy. Women who are breastfeeding or receiving one or more drugs with the potential to interact with the oral azoles should also be treated topically, as should those with a history of adverse reactions to the azoles or with significant liver impairment.

Whichever form of therapy is ultimately chosen, the shortest feasible course of treatment is advisable. The potential for resistance and pathogen shift with oral drugs argues for the use of broad-spectrum topical therapy.

Because the potential risks and disadvantages associated with oral therapy tend to outweigh the benefits, treatment with topical agents should be regarded as the method of choice for patients with VVC, although oral agents may have a place in the treatment of RVVC or in those patients in whom the benefits may outweigh any possible adverse reactions.

### REFERENCES

1. Sobel JD: Candidal vulvovaginitis. Clin Obstet Gynecol 36:153–165, 1993.
2. Horowitz BJ, Giaquinta D, Ito S: Evolving pathogens in vulvovaginal candidiasis: Implications for patient care. J Clin Pharmacol 32:248–255, 1992.
3. Corson SL, Kapikian RR, Nehring R: Terconazole and miconazole cream for treating vulvovaginal candidiasis. A comparison. J Reprod Med 36:561–567, 1991.
4. Osser S, Haglund A, Westrom L: Treatment of candidal vaginitis. A prospective randomized investigator-blind multicenter study comparing topically applied econazole with oral fluconazole. Acta Obstet Gynaecol Scand 70: 73–78, 1991.
5. Horowitz BJ: Mycotic vulvovaginitis: A broad overview. Am J Obstet Gynecol 165:1188–1192, 1991.
6. Redondo-Lopez V, Lynch M, Schmitt C, Cook R, Sobel JD: *Torulopsis glabrata* vaginitis: Clinical aspects and susceptibility to antifungal agents. Obstet Gynecol 76:651–655, 1990.
7. Boag FC, Houang ET, Westrom R, McCormack SM, Lawrence AG: Comparison of vaginal flora after treatment with a clotrimazole 500 mg vaginal pessary or a fluconazole 150 mg capsule for vaginal candidosis. Genitourin Med 67:232–234, 1991.
8. Ernest JM: Topical antifungal agents. Obstet Gynecol Clin North Am 19:587–607, 1992.
9. Nixon SA: Vulvovaginitis: The role of patient compliance in treatment success. Am J Obstet Gynecol 165:1207–1209, 1991.
10. Merkus JMWM: Treatment of vaginal candidiasis: Orally or vaginally? J Am Acad Dermatol 23:568–572, 1990.
11. Sobel JD: Pathogenesis and treatment of recurrent vulvovaginal candidiasis. Clin Infect Dis 14(Suppl 1):S148–S153, 1992.
12. Ng TTC, Denning DW: Fluconazole resistance in *Candida* in patients with AIDS—A therapeutic approach. J Infect Dis 26:117–125, 1993.
13. Scudamore JA, Tooley PJ, Allcorn RJ: The treatment of acute and chronic vaginal candidosis. Br J Clin Pract 46:260–263, 1992.
14. Stein GE, Mummaw N: Placebo-controlled trial of itraconazole for treatment of acute vaginal candidiasis. Antimicrob Agents Chemother 37:89–92, 1993.
15. Patel HS, Peters MD II, Smith CL: Is there a role for fluconazole in the treatment of vulvovaginal candidiasis? Ann Pharmacother 26:350–353, 1992.
16. Fong IW: The value of chronic suppressive therapy with itraconazole versus clotrimazole in women with recurrent vaginal candidiasis. Genitourin Med 68:374–377, 1992.
17. Odds FC: Resistance of yeasts to azole-derivative antifungals. J Antimicrob Chemother 31:463–471, 1993.
18. Del Palacio A: Fungal skin and soft tissue infections. Curr Opin Infect Dis 5:687–694, 1992.
19. Timonen H: Shorter treatment for vaginal candidosis: Comparison between single-dose oral fluconazole and three-day treatment with local miconazole. Mycoses 35: 317–320, 1992.
20. Hay RJ: Risk/benefit ratio of modern antifungal therapy: Focus on hepatic reactions. J Am Acad Dermatol 29:S50–S54, 1993.
21. Baciewicz AM, Baciewicz FA Jr: Ketoconazole and fluconazole drug interactions. Arch Intern Med 153:1970–1976, 1993.
22. 1994 Physician's Desk Reference. Montvale, NJ: Medical Economics Data Production Company, 1994.
23. Tett S, Carey D, Lee HS: Drug interactions with fluconazole. Med J Aust 156:365, 1992.
24. Clark C, Cooper CL, Gordon SF, van Amerongen D, Smith FO, Upmalis DH: A multicenter comparison of one-dose ticonazole ointment with three-dose terconazole cream in vulvovaginal candidiasis. J Women's Health 2:189–196, 1993.
25. Stein GE, Christensen S, Mummaw N: Comparative study of fluconazole and clotrimazole in the treatment of vulvovaginal candidiasis. Drug Intelligence and Clinical Practice 25:582–585, 1991.
26. Brammer KW, Feczko JM: Single-dose oral fluconazole in the treatment of vaginal candidiasis. Ann NY Acad Sci 554:561–563, 1988.
27. Abbott M, Hughes DL, Patel R, Kinghorn GR: Angiooedema after fluconazole. Lancet 338:633, 1991.
28. Neuhaus G, Pavcic N, Pletscher M: Anaphylactic reaction after oral fluconazole. Br Med J 302:1341, 1991.
29. Tobin JM, Loo P, Granger SE: Treatment of vaginal candidosis: A comparative study of the efficacy and acceptability of itraconazole and clotrimazole. Genitourin Med 68:36–38, 1992.
30. Slavin MB, Benrubi GI, Parker R, Griffin CR, Magee MJ: Single dose oral fluconazole vs. intravaginal terconazole in treatment of candida vaginitis. Comparison and pilot study. J Fla Med Assoc 79:693–696, 1992.
